# Population pharmacokinetic analysis and dosage recommendations for digoxin in Japanese patients with atrial fibrillation and heart failure using real-world data

**DOI:** 10.1186/s40360-022-00552-y

**Published:** 2022-02-10

**Authors:** Toshinori Hirai, Hidefumi Kasai, Miyoko Naganuma, Nobuhisa Hagiwara, Tsuyoshi Shiga

**Affiliations:** 1 Department of Clinical Pharmacology and Therapeutics, The Jikei University School of Medicine, 3-25-8 Nishi-shinbashi, Minato-ku, Tokyo, 105-8461 Japan; 2 Department of Pharmacy, Faculty of Medicine, Mie University Hospital, Mie University, Tsu, Japan; 3 Keio University School of Medicine, Tokyo, Japan; 4 Department of Pharmacy, International University of Health and Welfare Atami Hospital, Atami, Japan; 5 Department of Cardiology, Tokyo Women’s Medical University, Tokyo, Japan

**Keywords:** Atrial fibrillation, Amiodarone, Creatinine clearance, Digoxin, Heart failure, Population pharmacokinetics

## Abstract

**Background:**

Digoxin is an important treatment option for reducing the ventricular rate in patients with atrial fibrillation (AF) and heart failure (HF). Digoxin has a narrow therapeutic window and large interindividual variability. A low target blood concentration, especially ≤0.9 ng/mL, is recommended for patients with HF who are taking digoxin. This study aimed to develop a population pharmacokinetic model and to identify clinical factors that affect digoxin exposure and an optimal digoxin dosing regimen in Japanese patients with AF and HF.

**Methods:**

A population pharmacokinetic analysis was performed by using a nonlinear mixed effects model based on 3465 concentration points from 391 patients (>18 years) who were receiving oral digoxin. Using trough serum digoxin concentrations and clinical data, a population pharmacokinetic model was developed for determining covariates of clearance. A 1-compartment model was used to examine the interindividual variability of the oral clearance (CL/F) of digoxin. An appropriate dosage of digoxin was identified using Monte Carlo simulation.

**Results:**

The final model demonstrated that creatinine clearance (CL_CR_) and the use of amiodarone were factors that contributed to the CL/F of digoxin. Monte Carlo simulation results showed that with a daily maintenance dose of 0.25 mg, the intoxication risk window of a trough serum concentration of ≥0.9 ng/mL could be reached in more than half of patients regardless of renal function category or concurrent use of amiodarone. The appropriate maintenance dosage was 0.125 mg daily for most Japanese patients with AF and HF. However, with a daily dose of 0.125 mg, a trough serum concentration of ≥0.9 ng/mL could be reached in more than half of patients with renal impairments (CL_CR_ 30 mL/min) or concurrent use of amiodarone. A daily maintenance dose of 0.0625 mg was acceptable for these patients.

**Conclusions:**

CL_CR_ and the use of amiodaron were found to contribute to digoxin clearance using a population pharmacokinetic methodology. For Japanese patients with AF and HF, 0.125 mg is an appropriate daily digoxin maintenance dose, but a dose reduction is required for patients with CL_CR_ <30 mL/min or concurrent amiodarone use.

**Supplementary Information:**

The online version contains supplementary material available at 10.1186/s40360-022-00552-y.

## Background

Digoxin is an important treatment option for reducing the ventricular rate in patients with atrial fibrillation (AF) and heart failure (HF) although it is recommended as a second-line treatment [1, 2]. Digoxin has sympathoinhibitory and vagomimetic effects, which delay atrioventricular nodal conduction, leading to a reduction in the ventricular rate, as well as a positive inotropic effect [3]. The effect of digoxin on rate control with no deterioration of haemodynamic status may be appropriate for AF that is associated with systolic HF. However, adverse outcomes of digoxin limit its benefit in practice [4, 5].

Digoxin has a narrow therapeutic window [6]. High blood digoxin concentrations are a risk factor for digitalis intoxication [7–10], and the incidence of digitalis intoxication can be decreased when the digoxin dosage is adjusted based on blood digoxin concentrations [11, 12]. Moreover, a high blood digoxin concentration (≥1.2 ng/mL) is reported to be associated with a risk of mortality in patients with HF and even in patients with AF [13, 14]. Therefore, a low target blood concentration is now recommended for patients who are taking digoxin, especially ≤0.9 ng/mL, which is preferable for systolic HF [15].

Digoxin shows interindividual variability in pharmacokinetics, with a large volume distribution, mainly skeletal muscle distribution, and renal elimination through glomerular filtration and tubular secretion [16]. Various factors, including renal function and concurrent use of P-glycoprotein inhibitors, such as amiodarone and verapamil, have been reported to impact digoxin renal clearance [6, 17]. Because Japanese individuals show smaller body weights and decreased creatinine generation compared to individuals in the US and Europe [18], dosage adjustment is required based on a pharmacokinetic model of Japanese parameters. There have been no reports on dosage adjustment of digoxin in Japanese patients with AF and HF for whom digoxin is indicated. Population pharmacokinetics is a method of expressing pharmacokinetic properties in a target population from clinical data, including blood drug concentration, which involves estimation using nonlinear mixed-effects models that were developed by Sheiner and Beal [19, 20]. The present study aimed to develop a population pharmacokinetic model and to identify clinical factors that affect digoxin exposure and an optimal maintenance digoxin dosing regimen in patients with AF and HF.

## Methods

### Subjects

We conducted a cohort study of 391 consecutive patients with AF and HF aged 18 years and older who were taking oral digoxin at Tokyo Women’s Medical University Hospital between January 2008 and December 2016. To identify patients who were prescribed digoxin and in whom digoxin serum concentrations were measured, we first searched the automated outpatient accounting databases. Then, we confirmed that the identified patients had been diagnosed with AF and HF by checking medical records. HF was defined according to the American College of Cardiology/American Heart Association criteria [21]; the patients in our study were designated as stage C (current or prior symptoms of HF) or stage D (refractory HF). We excluded patients who were receiving methyldigoxin or patients whose trough concentration of digoxin was not measured. Details of the study design and data collection have been previously reported [10]. The protocol was approved by the institutional review boards of Tokyo Women’s Medical University and Jikei University.

### Data collection

The collected data from electronic medical records included demographic data (sex, age, height, body weight, and body mass index), left ventricular ejection fraction, New York Heart Association (NYHA) functional class, underlying heart disease, clinical laboratory data (serum creatinine), and data on digoxin (dosage and trough serum concentration) and concurrent potent P-glycoprotein inhibitor medications (amiodarone, diltiazem, and verapamil). Data collection covered the period between the initiation of oral digoxin therapy and the last measurement of the digoxin trough serum concentration or January 31, 2018. Renal function was assessed using the creatinine clearance (CL_CR_) and the estimated glomerular filtration rate (eGFR). CL_CR_ was calculated with the Cockcroft-Gault equation [22]. The eGFR was calculated with the Japanese version of the Modification of Diet in Renal Disease formula [18]. The serum digoxin concentration was assayed using the COBAS TDM system (Roche Diagnostics K.K., Tokyo, Japan) by the kinetic interaction of microparticles in a solution until November 2016. The detection limit of this assay was 0.3 ng/mL. The standard curves for digoxin were linear from 0.3 to 5.0 ng/mL. After that, the serum digoxin concentration was measured using the Nanopia TDM system (SEKISUI MEDICAL CO., LTD, Tokyo, Japan) by latex coagulating nephelometry. This assay had a detection limit of 0.05 ng/mL. The standard curves for digoxin were linear from 0.2 to 5.0 ng/mL. Digoxin concentrations that were measured at least 6 h after the last administration were used to develop a population pharmacokinetic model of the trough serum concentration [15]. All trough concentrations were regarded as steady-state concentrations because the trough concentrations were measured 5 days after the start of digoxin administration.

### Population pharmacokinetic model development

The population pharmacokinetic model was developed with a nonlinear mixed effects model using Phoenix NLME™ software (version 8.1, Certara USA, Inc., Princeton, NJ, USA). The base model was a one-compartment model with first-order absorption. Serum concentrations below measurable limits were not used to develop the population pharmacokinetic model. Because we could not obtain serum digoxin concentrations except for trough concentrations, the absorption rate constant was fixed to 1.0 h^− 1^ to reflect the assumption that digoxin was absorbed fast irrespective of P-glycoprotein function [23]. In addition, the apparent volume of distribution (Vd) was fixed to 6.0 L/kg according to a previous study [24]. Because we could not estimate the absolute bioavailability, the oral clearance (CL/F) was estimated. The intraindividual variability was compared using an additive error model and a multiplicative error model, which were defined as follows:

$${C}_{obs}={C}_{pred}+\varepsilon$$

$${C}_{obs}={C}_{pred}\times \left(1+\varepsilon \right)$$

where C_obs_ and C_pred_ denote the observed and predicted serum digoxin concentrations, respectively, and ε denotes the measurement error, which includes the intraindividual variability, analytical error, and dosing error. We assessed using the objective function value. The difference of 3.84 in the objective function value between these models was statistically significant (*p* < 0.05).

The interindividual variability of the oral clearance of digoxin was described using an exponential random effects model, which was defined as follows:

$$CL/F= tv\ CL/F\times {e}^{\eta }$$

where CL/F denotes the parameter for digoxin oral clearance, tv CL/F is the typical value of oral clearance and η represents the interindividual variability of CL/F. We used a stepwise forward selection method to assess the impacts of covariates on the CL/F of digoxin. The potential covariates were demographic data (sex, age, and body mass index), renal function (CL_CR_ and eGFR), and concurrent medications (amiodarone, diltiazem, and verapamil). Continuous covariates were normalized by their typical values. If CL_CR_ was estimated to be above 120 mL/min, it was replaced with 120 mL/min to avoid the overestimation of renal clearance. Sex and concurrent medications were regarded as categorical covariates. Potential covariates were incorporated one by one into the base model and assessed using the value of the objective function that is mentioned above. Initially, potential covariates that produced the minimum value of the objective function were screened and added to the base model. After the selection of a potential covariate, we explored whether the addition of this potential covariate improved the model performance in the same manner. If we detected multicollinearity of covariates, we chose a covariate according to both the minimum value of the objective function and the clinical relevance.

### Population pharmacokinetic model evaluation

We evaluated the fit and robustness of the final model using goodness-of-fit plots and bootstrap methods. The final model fit was evaluated by scatter plots of observed vs. predicted concentrations, observed vs. individual predicted concentrations, conditional weighted residuals vs. predicted concentrations, and conditional weighted residuals vs. time after the first dose. Using a bootstrap method, 1000 samples were generated by random resampling of the original dataset. The final model parameters of the 1000 generated samples were compared to those from the original dataset.

### Simulations

Monte Carlo simulation was performed every 1000 iterations using the final model to identify an optimal dosing regimen at various daily doses (0.25 mg, 0.125 mg, and 0.0625 mg). This simulation was performed using a lognormal distribution based on the interindividual variation that was obtained by population pharmacokinetic analysis. Predicted serum digoxin concentrations were compared according to CL_CR_ (90 mL/min, 60 mL/min, and 30 mL/min) and with or without concurrent use of amiodarone. Serum digoxin concentrations of ≥0.9 ng/mL and ≥1.2 ng/mL were defined as trough concentrations above the target range because these cut points of clinical interest were used in previous studies of patients with HF [13, 25]. We calculated the probabilities of trough serum digoxin concentrations being ≥0.9 ng/mL and ≥1.2 ng/mL.

### Statistical analysis

Continuous data are presented as the mean ± standard deviation (SD) for those with a normal distribution or as the median and interquartile range for those with a nonnormal distribution unless otherwise specified. Categorical data are presented as numerical values (%). Data analyses were performed using JMP Pro statistical software (version 14, SAS Institute Inc., Cary, NC, USA).

## Results

### Study population

The clinical features of the study population are listed in Table 1. The mean age was 67 ± 14 years, and the median CL_CR_ was 56.5 [40.7–75.6] mL/min. Among the 391 patients, 312 (80%) had permanent/persistent AF, and 100 (26%) were of NYHA functional class III or IV. Regarding underlying heart disease, nonischaemic aetiologies, including cardiomyopathies and valvular disease were common in our patients. Approximately 70% of patients received a daily dose of 0.125 mg digoxin. The median treatment duration was 350 [60–1340] days. Amiodarone was the most common concurrent P-glycoprotein inhibitor.

**Table 1:** Patient characteristics (*n* = 391)

Variable	
Demographic data	
Female, n (%)	148 (38)
Age, years	67 ± 14
Height, cm	161 ± 11
Body weight, kg	57 ± 15
Body mass index, kg/m^2^	22 ± 4
LVEF, %	39 ± 14
NYHA class II/III/IV, n	291/67/33
Underlying heart disease	
Coronary artery disease, n (%)	59 (15)
Nonischaemic cardiomyopathies, n (%)	109 (28)
Valvular disease, n (%)	57 (15)
Hypertensive heart disease, n (%)	27 (7)
Congenital heart disease, n (%)	26 (7)
Others, n (%)	113 (29)
Atrial fibrillation	
Permanent/persistent, n (%)	312 (80)
Paroxysmal, n (%)	76 (20)
ICD/pacemaker, n (%)	53 (14)
Clinical laboratory data	
Serum creatinine, mg/dL	0.94 [0.75–1.15]
CL_CR_, mL/min	56.5 [40.7–75.6]
eGFR, mL/min/1.73m^2^	58.1 [44.7–71.0]
Daily dose of digoxin	
0.25 mg, n (%)	51 (13)
0.125 mg, n (%)	287 (73)
0.0625 mg, n (%)	39 (10)
Other doses, n (%)	14 (4)
Duration of digoxin treatment, days	350 [60–1340]
Concurrent medications of interest	
Amiodarone, n (%)	63 (16)
Diltiazem, n (%)	33 (8)
Verapamil, n (%)	23 (6)

Values are n (%) or means ± SD or median [interquartile range]

*CL*_*CR*_ creatinine clearance, *eGFR* estimated glomerular filtration rate, *ICD* implantable cardioverter-defibrillator, *LVEF* left ventricular ejection fraction, *NYHA* New York Heart Association

Regarding clinical outcomes, the relationships between the events and values of digoxin concentration immediately after or before (within 3 months) the occurrence of the events are presented in Table 2. Regarding the cause of death, the incidence of cardiac death did not increase with increased serum concentrations of digoxin. The incidence of noncardiac death increased in patients with serum digoxin concentration of ≥0.90 ng/mL. Regarding the type of digoxin intoxication that occurred, cardiac disturbance was observed in all of the concentration groups, whereas gastrointestinal symptoms were observed only in patients with serum digoxin concentrations of ≥0.90 ng/mL.

**Table 2:** Summary of outcomes and serum digoxin concentration

Serum digoxin concentration, ng/mL	<0.60	0.60–0.89	0.90–1.19	≥1.20
Death	5	7	6	4
Cause of death				
Heart failure	1	2	1	1
Sudden cardiac death	1	3	1	0
Noncardiac causes	3	2	4	3
Digoxin intoxication	3	1	5	8
Type of digoxin intoxication				
Cardiac disturbance	2	1	4	3
Gastrointestinal symptoms	0	0	1	4
Others	1	0	0	1

Values are n

### Population pharmacokinetic model development

A total of 3465 digoxin trough serum concentrations from 391 patients were used in the following analysis. At the first measurement of the trough digoxin concentration, the median serum concentration was 0.77 [0.52–1.02] ng/mL. The median number of measurements for each patient was 5 [2–12].

A one-compartment model and first-order absorption with a multiplicative error model were found to best describe the trough serum digoxin concentrations. The stepwise forward selection method identified the following potential covariates of CL/F: body mass index, CL_CR_, eGFR, use of amiodarone, and use of diltiazem. Among them, CL_CR_ produced the minimum objective function value and was included in the base model. After adding CL_CR_ to the base model, concurrent use of amiodarone yielded the maximum reduction of the objective function value, which was significant. There remained no clear relationships in the final model between the random effect for CL/F and other covariates, such as sex, age, height, and body mass index (Supplemental Fig.). Thus, CL_CR_ and concurrent use of amiodarone were established as covariates of CL/F and included in the final model of digoxin (Table 3).

**Table 3:** Final population pharmacokinetic model estimates and bootstrap results

Parameter	Estimated mean	RSE %	Bootstrap mean	95% CI
Population value				
CL/F, L/h	6.209	2.830	6.215	6.207–6.223
CL_CR_ on CL/F	0.409	9.491	0.415	0.412–0.417
Amiodarone on CL/F	−0.238	3.158	−0.243	−0.246 – − 0.241
Vd/F, L/kg (fixed)	6.000	NA	6.000	NA
k_a_, h^−1^ (fixed)	1.000	NA	1.000	NA
Interindividual variability				
ωCL/F, %	34.4	2.9	34.2	34.1–34.4
Intraindividual variability				
Multiplicative, %	36.6	3.2	36.5	34.3–38.9

*CI* confidence interval, *CL*_*CR*_ creatinine clearance, *CL/F* oral clearance, *k*_*a*_ absorption rate constant, *NA* not available, *RSE* relative standard error, *Vd/F* apparent volume distribution

The final model parameters were as follows

*CL*/*F* = 6.2 × (*CLcr*/60)^0.41^ × (1 − 0.24 × [*if amiodarone*])

*Vd*/*F* = 6.0 × *Body weight*

*k*_*a*_ = 1.0

The estimated mean oral clearance (relative standard error, %) was 6.2 L/h (2.8%). The final model for CL/F was defined as follows:

$$CL/F=6.2\times {\left({CL}_{CR}/60\right)}^{0.41}\times \left(1-0.24\times \left[ if\ amiodarone\right]\right)$$

where 0.41 is the exponential coefficient for CL_CR_ and 0.24 is the fractional change (decrease) for concurrent use of amiodarone.

### Population pharmacokinetic model evaluation

**Fig. 1:**
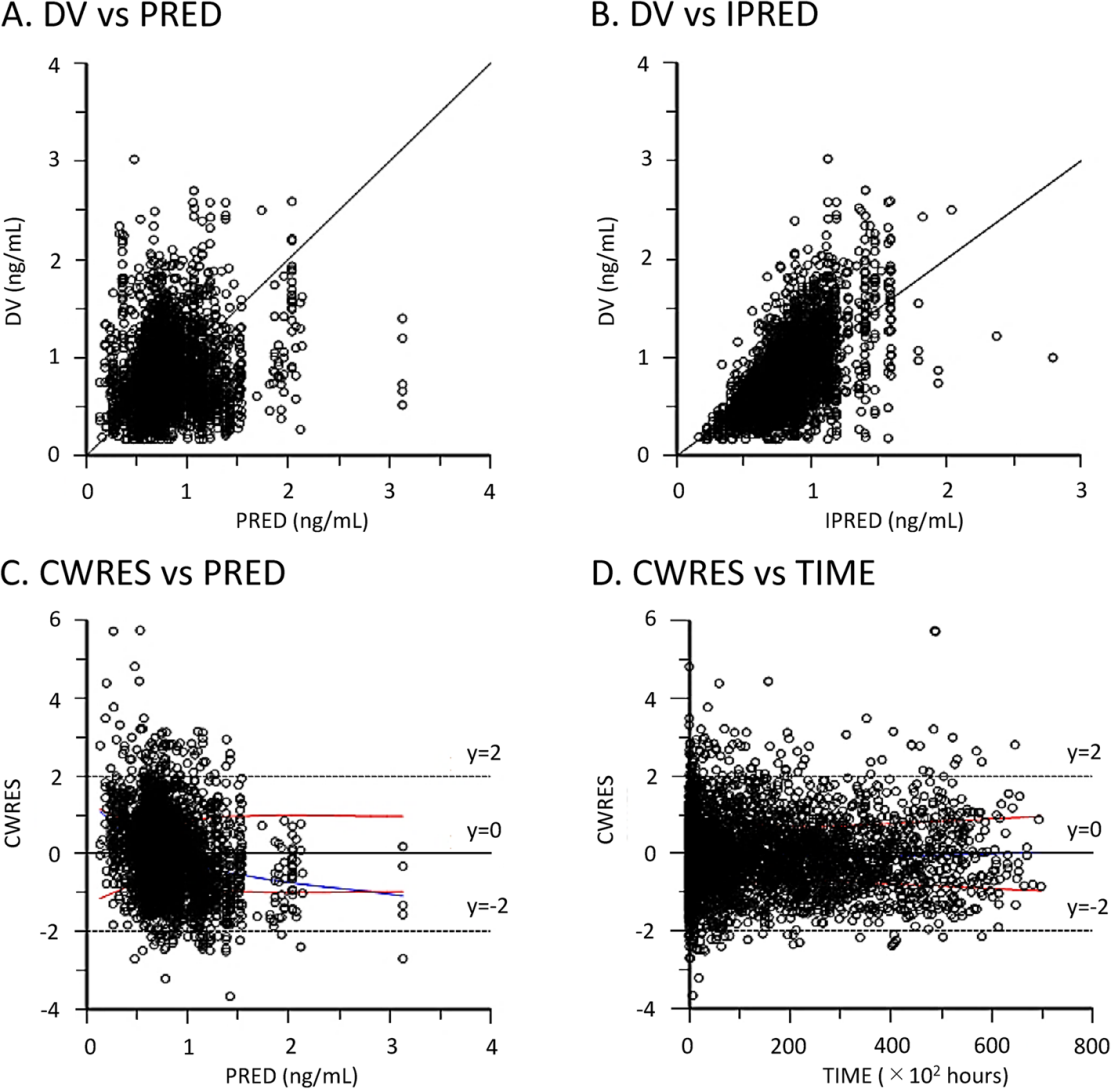
Goodness-of-fit scatter plots for the final population pharmacokinetic model. **A**. Observed concentrations (DV) vs. predicted concentrations (PRED); the solid line represents the reference line. **B**. Observed concentrations (DV) vs. individual predicted concentrations (IPRED); the solid line represents the reference line. **C**. Conditional weighted residuals (CWRES) vs. predicted concentrations (PRED); the solid and dotted horizontal lines represent the reference line and ± 2 standard deviations, respectively. The red curve is a loess curve fit to the absolute values of the residuals. The bottom red curve is the reflection of the top red curve about the x-axis. The blue line is the loess curve fit to the raw residuals. **D**. Conditional weighted residuals (CWRES) vs. time after the first dose (TIME); the solid and dotted lines represent the reference line and ± 2 standard deviations, respectively. The red curve is a loess curve fit to the absolute values of the residuals. The bottom red curve is the reflection of the top red curve about the x-axis. The blue line is the loess curve fit to the raw residuals

Scatter plots of observed vs. predicted concentrations (Fig. 1A), observed vs. individual predicted concentrations (Fig. 1B), conditional weighted residuals vs. predicted concentrations (Fig. 1C), and conditional weighted residuals vs. time after the first dose (Fig. 1D) did not show any systematic bias.

The success rate of the bootstrap method was 100%, and the mean bootstrap parameters were close to the estimated values that were obtained from the original dataset (Table 3).

### Simulation

**Fig. 2:**
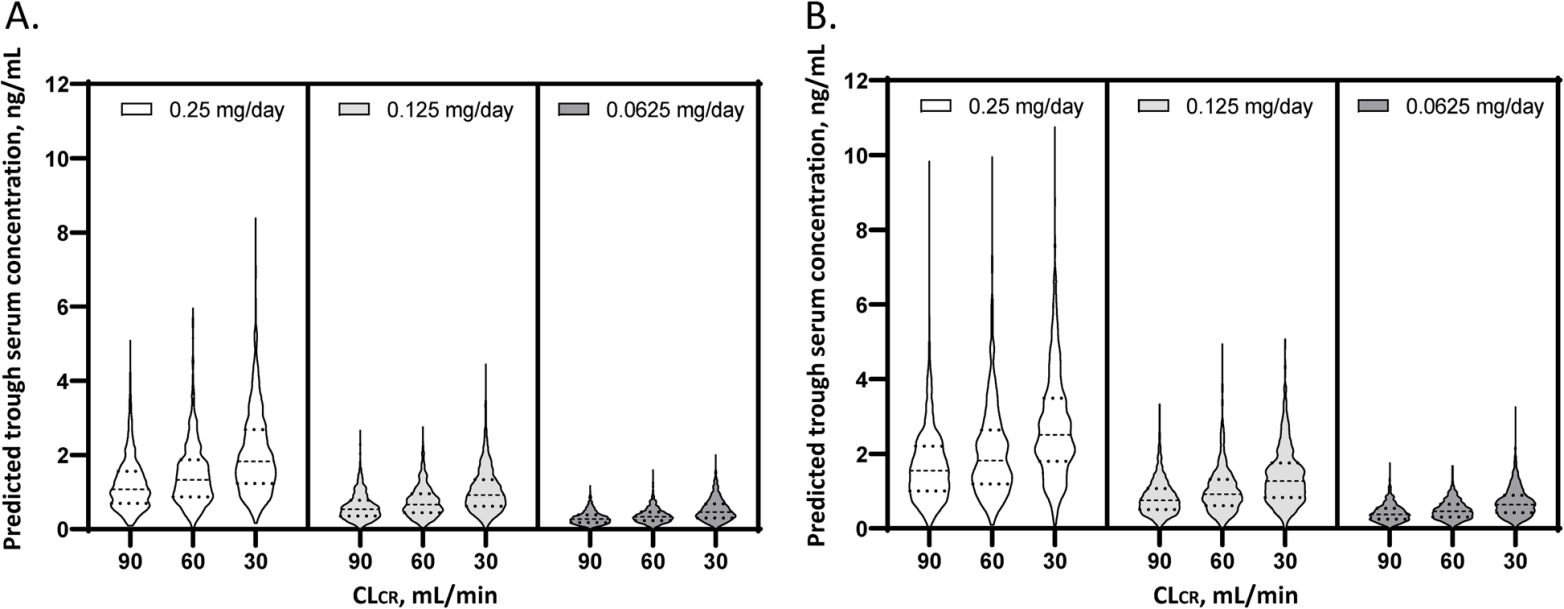
Summary of the Monte Carlo simulation. **A**. Violin plots for the predicted trough serum concentration according to the digoxin dosage and creatinine clearance in the absence of amiodarone. **B**. Violin plots for the predicted trough serum concentration according to the digoxin dosage and creatinine clearance in the presence of amiodarone. The dashed and dotted lines represent the median and interquartile range, respectively. The violin plots indicate the distributions of the predicted digoxin trough serum concentration according to the dosage

The Monte Carlo simulations are summarized in Table 4 and Fig. 2. A daily maintenance dose of 0.25 mg could reach the intoxication risk window of a trough serum concentration of ≥1.2 ng/mL in nearly half of patients and ≥0.9 ng/mL in more than half of patients, regardless of renal function category or concurrent use of amiodarone. A daily maintenance dose of 0.125 mg could not be tolerated in patients with renal impairments (CL_CR_ 30 mL/min) or concurrent use of amiodarone because more than half of patients reached a trough serum concentration of ≥0.9 ng/mL. A daily maintenance dose of 0.0625 mg was appropriate as a maintenance dose for patients with renal impairments or concurrent use of amiodarone.

**Table 4:** Probabilities of digoxin concentration reaching intoxication risk ranges according to renal function and the use of amiodarone

Daily maintenance dose of digoxin, mg	CL_CR_, mL/min	No amiodarone		Amiodarone	
		≥0.9 ng/mL	≥1.2 ng/mL	≥0.9 ng/mL	≥1.2 ng/mL
0.25	90	61.9	43.0	79.0	65.7
0.25	60	72.7	55.7	86.4	74.5
0.25	30	86.9	76.3	95.0	88.1
0.125	90	18.7	7.0	37.1	19.0
0.125	60	28.1	12.4	51.6	31.5
0.125	30	51.3	31.1	71.4	54.4
0.0625	90	0.9	0.0	3.7	0.7
0.0625	60	1.8	0.5	8.2	1.7
0.0625	30	11.2	3.1	24.3	10.6

Values are percentages (numbers of patients)

*CL*_*CR*_ creatinine clearance

## Discussion

We developed a population pharmacokinetic model of digoxin in Japanese patients with AF and HF using real-world data. This population pharmacokinetic analysis identified that CL_CR_ and concurrent use of amiodarone influenced digoxin clearance and the appropriate maintenance dosage was 0.125 mg daily for almost all Japanese patients with AF and HF. However, 0.0625 mg daily appeared to be a suitable dosage for patients with renal impairments (CL_CR_ 30 mL/min) or concurrent use of amiodarone.

Digoxin has a large Vd and ethnic differences in Vd that are corrected by kilograms of body weight have not been reported. We used the previously reported value of digoxin Vd [24] in this model because it does not influence the trough serum concentration at the steady state. Digoxin is excreted primarily by the kidney (≥70%), and renal excretion of digoxin is correlated with the glomerular filtration rate [6]. Previous studies have shown that CL_CR_ is associated with the clearance of digoxin [26–28]. Because the Cockcroft-Gault formula is based on serum creatinine, body weight, age, and sex [22], these factors also influence the clearance of digoxin [29]. In this study, CL_CR_ was established as the most useful covariate for the clearance of digoxin among several covariates. CL_CR_ is not adjusted for body surface area and may more comprehensively reflect the individual situation.

Our results also showed that the use of amiodarone was a significant covariate of the clearance of digoxin. Digoxin is a substrate of P-glycoprotein, which contributes to its absorption and elimination [17]. Several medications are known to interact with digoxin via P-glycoprotein and affect the pharmacokinetics of digoxin [30]. In particular, amiodarone has a relatively high inhibition capacity against P-glycoprotein [30]. It has been reported that high-dose amiodarone administration (600 to 1600 mg daily) doubles digoxin plasma concentrations [31]. In this study, the effect of dose-dependent inhibition of amiodarone on digoxin pharmacokinetics was not clarified.

Our Monte Carlo simulation results indicated that 0.25 mg daily of digoxin was an unacceptable dosage because an estimated serum concentration of ≥1.2 ng/mL was predicted to occur in more than half of patients with AF and HF. Miura et al. reported that digoxin intoxication was observed even in Japanese patients with AF and/or HF whose serum concentrations were between 1.4 and 2.0 ng/mL [32]. We previously demonstrated that a serum concentration of ≥1.2 ng/mL was significantly associated with an increased risk of digoxin intoxication in patients with AF and HF [10]. The present analysis showed that 0.125 mg daily of digoxin is a favourable maintenance dosing regimen for a large proportion of Japanese patients with AF and HF. In patients with CL_CR_ <30 mL/min or concurrent use of amiodarone, however, 0.125 mg daily is an undesirable dosing regimen. Dosage reduction to 0.0625 mg daily is recommended for these patients to avoid a poor prognosis and digoxin intoxication. Komatsu et al. suggested that an extremely low dosage of digoxin, namely, 0.0625 mg daily, was also suitable for Japanese patients with CL_CR_ <35 mL/min or concurrent use of amiodarone but that 0.1875–0.25 mg daily was recommended for patients with CL_CR_ >60 mL/min when the target serum digoxin concentration range was 0.5–0.8 ng/mL [33]. This discrepancy might be due to differences in the clinical characteristics of patients. The presence of HF is reported to influence the clearance of digoxin [29]. Renal clearance of digoxin decreases in patients with HF compared to patients without HF despite no difference in digoxin dosage, creatinine clearance, diuresis, or sodium excretion in the urine [34]. Therefore, the blood concentration of digoxin is higher in patients with HF than in patients without HF. Approximately half of Japanese patients with HF have renal impairment, and 10% receive amiodarone for complicated arrhythmias [35].

In patients with HF, renal clearance of a drug decreases because low cardiac output reduces renal blood flow and the glomerular filtration rate, in addition to causing renal parenchyma and renal tubule disorders (renal failure). Therefore, the clearance of digoxin is reduced, and the blood concentration is increased [34]. This study recommends dosages for patients with HF and also takes into account the effects of amiodarone, which is frequently used in patients with arrhythmias and HF, based on clinical data from patients with AF and HF and a reference target serum digoxin concentration range for patients with HF. In clinical practice, some patients required higher blood digoxin concentrations to maintain haemodynamics and adequate heart rate control. However, gastrointestinal complications increased with increased serum digoxin concentration among our patients. It is important to start with the recommended maintenance dosage and to adjust the dosage based on a thorough examination of the individual’s symptoms, effects, and side effects. We believe that our results will help ensure the safety of digoxin treatment for Japanese patients with AF and HF.

### Study limitations

This study has several limitations that should be considered in the interpretation of the results. First, because this study had a single-centre retrospective observational design, we could not avoid the possibility of bias. Due to the sample size, this study was limited to assessing the impact in a special population (e.g., individuals with obesity and undergoing renal replacement therapy). Adherence to pharmacotherapy could not be evaluated. Second, the method of measuring the samples that were analysed in this study changed in December 2016. Because the accuracies of the measurements did not differ significantly between the methods before and after the change and no clinical problems occurred due to change in the measurement method, the measurement values were used in this study regardless of their measurement method. Third, this study did not fully investigate all drugs that may pharmacokinetically interact with digoxin. Due to the small sample size of this study, we limited the considered drugs to P-glycoprotein inhibitors, such as amiodarone, verapamil and diltiazem, that are expected to be used in combination in patients with AF and HF. Fourth, this study included only patients who were treated at a single centre. There was also treatment bias. The clinical characteristics of our patients might not reflect those of general HF patients because our institution is a university hospital in the metropolitan Tokyo area. Therefore, the findings could not be generalized to all Japanese patients with HF. It is unclear whether the use of the recommended dosages would improve outcomes including, e.g., in term of digitalis intoxication and death, in general Japanese patients with AF and HF. To evaluate this, validation in another patient population from this study will be required.

## Conclusions

Our constructed population pharmacokinetic model indicates the clinical significance of CL_CR_ and the use of amiodaron for digoxin oral clearance. A low maintenance dosage of digoxin, namely, 0.125 mg daily, is appropriate for Japanese patients with AF and HF, and the dosage should be reduced to 0.0625 mg daily for patients with CL_CR_ <30 mL/min or concurrent use of amiodarone.

## Supplementary Information

**Additional file 1: Supplemental Fig.** Relationships between the random effect for CL (eta CL) and the potential covariates (sex, age, height and body mass index) in the final model.

## Data Availability

The datasets generated during and/or analysed during the current study are available from the corresponding author on reasonable request.

## Abbreviations

AF: Atrial fibrillation
CL_CR_: Creatinine clearance
CL/F: Oral clearance
eGFR: Estimated glomerular filtration rate
HF: Heart failure
Vd: Apparent volume of distribution
