# Supplementary material for: Population pharmacokinetic analysis and dosage recommendations for digoxin in Japanese patients with atrial fibrillation and heart failure using real-world data
Source: BMC Pharmacol Toxicol. 2022 Feb 10;23:14. doi: 10.1186/s40360-022-00552-y (PMC8830040; doi:10.1186/s40360-022-00552-y)
Supplement: Supplementary file 1 — Additional file 1: Supplemental Fig. Relationships between the random effect for CL (eta CL) and the potential covariates (sex, age, height and body mass index) in the final model. [file 40360_2022_552_MOESM1_ESM.pdf]

Population pharmacokinetic analysis and dosage recommendations for digoxin in Japanese patients with atrial fibrillation and heart failure using real-world data

Toshinori Hirai, Hidefumi Kasai, Miyoko Naganuma, Nobuhisa Hagiwara, Tsuyoshi Shiga

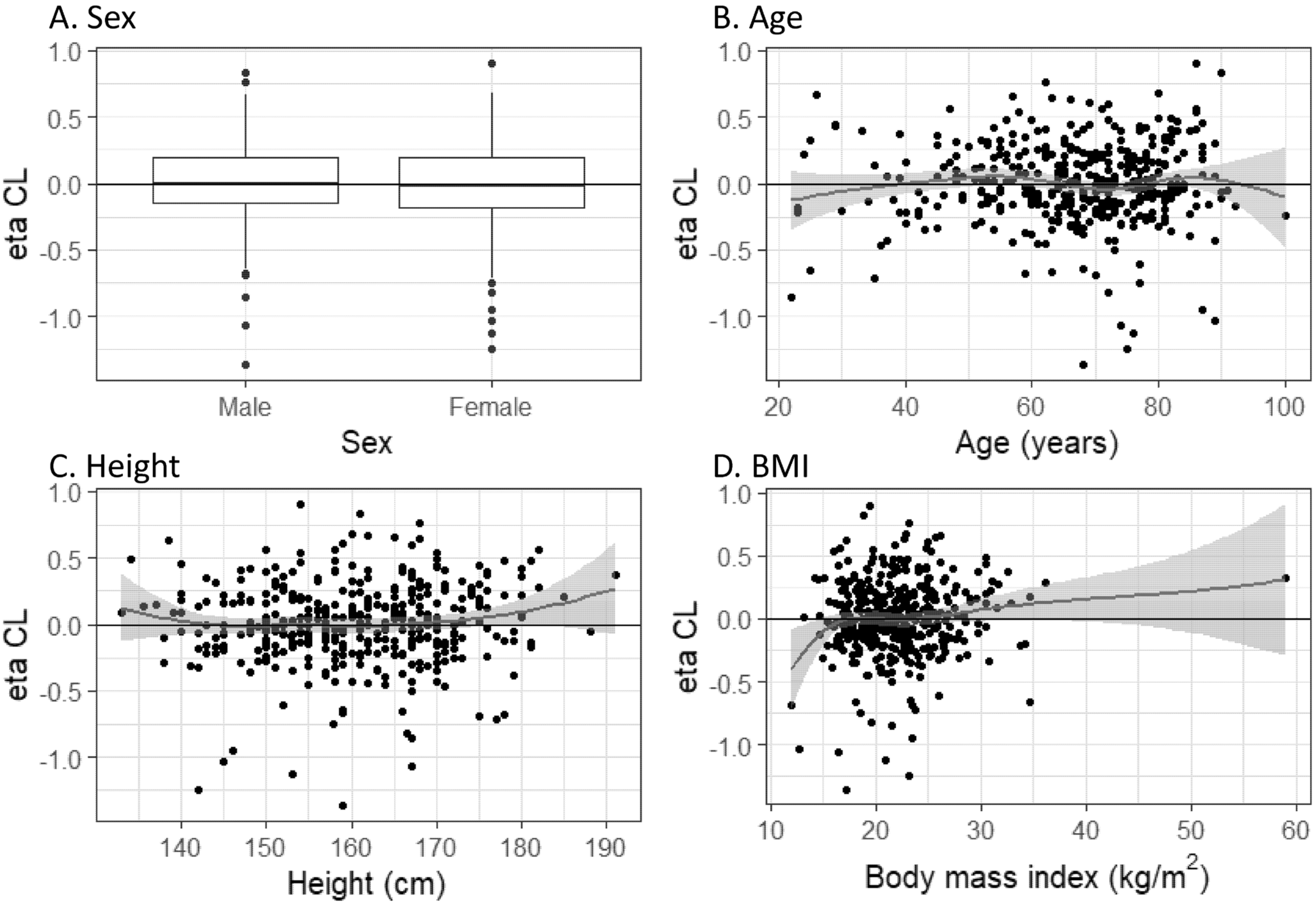

Supplemental Fig. Relationships between random effect for CL (eta CL) and the potential covariates in the final model.

A: A boxplot for eta CL by sex. The lower and upper edges of the box correspond to the first and third quartiles (the 25th and 75th percentiles). The upper whisker extends from the edge to the largest value no further than 1.5*IQR from the edge (where IQR is the interquartile range or distance between the first and third quartiles). The lower whisker extends from the edge to the smallest value at most 1.5*IQR from the edge. Data beyond the ends of the whiskers are plotted individually.

B, C, D: Scatter plots between eta CL and age (B), height (C), and body mass index (BMI) (D). The blue line in the plot presents the smoothing curve, and its 95% confidence interval is represented by a grey shadow.
